# The amygdala as a therapeutic target for aggressive and disruptive behaviors: a systematic review

**DOI:** 10.47626/1516-4446-2024-3582

**Published:** 2024-11-25

**Authors:** Juan Camilo Salcedo-Moreno, Fernando Peralta-Pizza, Paola Vélez-Jimenez, David Arteaga-Ortiz, Lina María Villegas-Trujillo, Oscar Andrés Escobar-Vidarte

**Affiliations:** 1 Sección de Neurocirugía, Universidad del Valle, Cali, Colombia; 2 Sección de Neurocirugía, Universidad Javeriana, Cali, Colombia; 3 Escuela de Medicina, Universidad del Valle, Cali, Colombia; 4 Sección de Neurocirugía, Hospital Universitario del Valle, Cali, Colombia; 5 Departamento de Neurocirugía, Fundación Valle del Lili, Cali, Colombia

**Keywords:** Aggressive behavior, stereotactic surgery, neuromodulation, amygdala.

## Abstract

**Objective:**

To identify the effects of amygdala neuromodulation on disruptive behavior and quality of life changes in patients and its relationship with epilepsy.

**Methods:**

The MEDLINE, OVID, WoS, Central Cochrane, and Scopus databases were systematically searched up to March 2023 for studies with at least six months of follow-up on extremely aggressive patients who underwent ablative surgeries or deep brain stimulation of the amygdala as the unique therapeutic target.

**Results:**

The search yielded 1,352 studies after excluding duplicates. However, only 11 case series and three case reports met the inclusion criteria. The studies were published between 1963 and 2023 and included 1,033 patients, mostly male, aged between 5 and 61 years. All of the studies performed amygdalotomy except one, which performed deep brain stimulation. Behavior improved in > 70% of the patients, and seizures occurred in approximately 30%, of whom 40% presented improvement. Two studies reported worsening behavior.

**Conclusions:**

Although we found that amygdalotomy has a positive effect on patient behavior and seizure control, new studies with greater power are needed. Future studies should investigate deep brain stimulation and the role of connectomics regarding this brain structure.

## Introduction

Aggressive behavior is an essential primitive survival response in many species in relation to food, territory, and reproduction.^1^ In humans, aggressive behavior is much more complex and can be classified in two subtypes. The first is premeditated aggressive acts, also called proactive aggressiveness, which are planned behaviors in pursuit of a specific objective. The second is acts triggered by impulsivity, instinct, and anger, also called reactive aggressiveness, which are usually associated with a traumatic event or stressor that varies in intensity across individuals.^1^ When the aggressive behavior is persistent and out of context, it is most often associated with some pathology and is treated as a symptom rather than an independent entity.^2^ Instrumental or premeditated behavior is more often associated with antisocial personality disorder, while reactivity, appetitive, or impulsive behavior is associated with epilepsy, congenital structural lesions or those secondary to infectious, hypoxic, or traumatic encephalopathy, or to psychiatric pathologies, such as intellectual disability, autism, schizophrenia, etc.^3^

The limbic system, an important brain region involved in aggressive behavior regulation, consists of multiple structures, including the amygdala, in a neural network that can interconnect afferent or efferent signals on multiple levels.^2^ This structure receives inhibitory signals from the frontal cortex, and, together with the hypothalamus, is one of the main areas related to aggressive behaviors in response to stressful stimuli.^2^ Its main function is to process threat signals and mediate a response, generally automatic and impulsive, to adapt to the environment.^1^

Based on neurobiological findings that extreme aggressive behavior is a symptom of several psychiatric pathologies and structural neurological disorders, it has been proposed that surgical intervention could be considered in cases refractory to pharmacological treatment.

Optogenetic studies in rodents and functional magnetic resonance imaging and positron emission tomography imaging studies in humans have shown that hyperactivity of the amygdala is associated with extreme aggressiveness. Thus, in the past 60 years, more than 1,000 amygdala ablation procedures have been performed.^1^,^4^-^6^ Many of these studies have suggested that ablation leads to higher tolerance to provocation and decreases autonomic arousal, thereby decreasing aggressive behavior.^1^,^7^,^8^ For this reason, we aimed to identify the effects of ablative therapy or deep brain stimulation (DBS) of the amygdala on intermittent explosive disorder and its relationship with the epileptic seizures typical of some of these patients.

## Methods

This systematic review was conducted in accordance with the Cochrane Manual for Systematic Reviews of Interventions^9^ and the 2020 Preferred Reporting Items for Systematic reviews and Meta-Analyses guidelines.^10^ The study protocol was preregistered in the OSF platform (https://osf.io/7jb3d/).

### Eligibility criteria

All case reports, case series, case-control studies, and controlled clinical trials of patients with intermittent explosive disorder who underwent amygdala ablation (stereotactic or neuromodulatory) were retrieved. Only studies with ≥ 6 months of follow-up were included. There were no restrictions on age group, sex, race, or publication date. We excluded all studies with any therapeutic target in addition to the amygdala.

### Information sources

The MEDLINE, OVID, WoS, Scopus and Central Cochrane databases were systematically searched up to March 2023. The search was conducted in English, and the basic search strategy was as follows (search equations used in each database can be found in Supplementary Box S1): ((TITLE-ABS-KEY (“aggressivity”) OR TITLE-ABS-KEY (“aggression”) OR TITLE-ABS-KEY (“aggressive behavior”) OR TITLE-ABS-KEY (“aggressive behavior”) OR TITLE-ABS-KEY (“aggressive behaviors”) OR TITLE-ABS-KEY (“behavioral disorders”) OR TITLE-ABS-KEY (“behavior disorders”) OR TITLE-ABS-KEY (“disruptive behavior”) OR TITLE-ABS-KEY (“violent behavior”) OR TITLE-ABS-KEY (“violent behavior”) OR TITLE-ABS-KEY (“explosive disorder”) OR TITLE-ABS-KEY (“aggressiveness”))) AND ((TITLE-ABS-KEY (“psychosurgery”) OR TITLE-ABS-KEY (“neurosurgical treatment”) OR TITLE-ABS-KEY (“stereotactic”) OR TITLE-ABS-KEY (“stereotaxic”) OR TITLE-ABS-KEY (“amygdalotomy”) OR TITLE-ABS-KEY (“neuronavigation”) OR TITLE-ABS-KEY (“radiosurgery”) OR TITLE-ABS-KEY (“amygdalectomy”) OR TITLE-ABS-KEY (“psycho surgery”) OR TITLE-ABS-KEY (“psycho surgery”) OR TITLE-ABS-KEY (“dbs”) OR TITLE-ABS-KEY (“deep brain stimulation”)).

### Study selection

The search results were reviewed by two evaluators who independently applied the inclusion and exclusion criteria. They initially screened the titles and abstracts, after which the full texts of the remaining studies were screened. At each selection stage, disagreements between the authors were resolved through discussion.

### Data collection process

The evaluators independently extracted the following data from the selected studies: author, year of publication, country, study design, number of participants, sex, age, procedure type, laterality, coordinates of the surgical target, surgical technique, adverse effects, aggressiveness evaluation scales, and follow-up time. The main outcome variables were behavior changes before and after the intervention (pre- and postoperative), the procedure’s impact on patient quality of life, and any seizure improvement in patients with epilepsy.

### Risk of bias assessment

The quality of the included series and case reports was assessed with an instrument developed by Murad et al.,^11^ which includes the following domains: 1) selection; 2) verification of exposure; 3) verification of the results; 4) causality; 5) follow-up duration; and 6) reports. Based on the study’s methodology, each domain was scored as 1 for positive responses or 0 for negative responses. Follow-up duration < 6 months was considered a negative response. Studies with zero or one negative responses overall were considered “good,” those with two or three negative responses were “acceptable,” and those with ≥ four negative responses were considered “deficient.”

### Statistical analysis

Normally distributed continuous quantitative variables were presented as means, standard deviations, and correlation measurements. Non-normally distributed variables were reported as medians and interquartile ranges. Categorical variables were reported as percentages, frequencies, and proportions.

## Results

### Study selection

**Figure 1:**
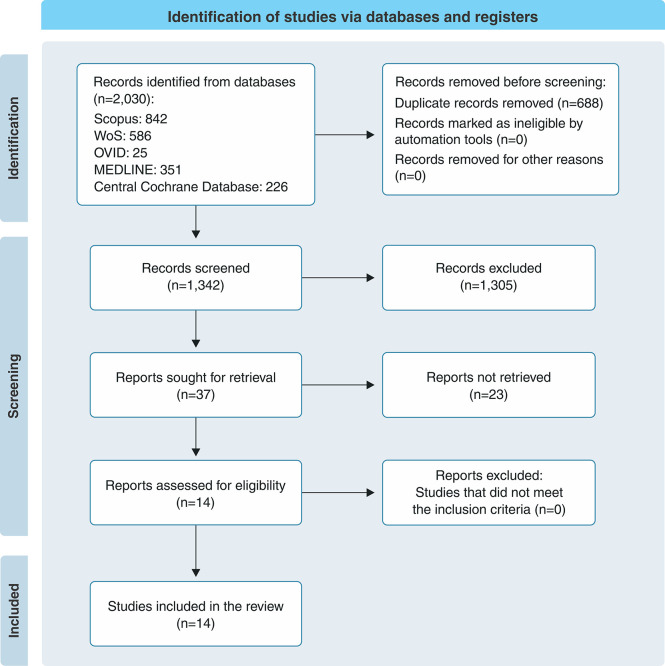
Study selection flowchart according to the 2020 Preferred Reporting Items for Systematic reviews and Meta-Analyses methodology.

The search returned a total of 1,352 records after duplicates were removed. A total of 11 case series and three case reports met the eligibility criteria and were included in the analysis (Figure 1). Table 1 shows the general characteristics of the included studies, which were primarily conducted in India, Japan, and the United States. The case series were published between 1963 and 2021, while the case reports were published in 1998, 2007, and 2012. A total of 1,033 patients were included and, although not all studies were organized by sex, approximately 70% of the patients were men. The patients’ ages ranged from 0 to 61 years.

Behavior improved in approximately 70% of patients, although in < 1% detrimental changes occurred. About 20% of the studies reported no changes in behavior. Approximately 30% of patients had concomitant epilepsy, of whom 40% showed improvement. The mean follow-up ranged from 6 months to 8 years. Quality of life improvement was measured by returning to school or work activities, deinstitutionalization, and improved social relationships (Table 2).

A bilateral procedure was performed in 90% of the patients, with different anatomical coordinates for the target. Surgical complications were reported in 40% of the articles, with an overall percentage < 1%.

### Study quality assessment

Each study chosen for this systematic review was carefully evaluated according to the previously-mentioned instrument.^11^ The studies’ quality scores are shown in Table 3. Most were classified as good or fair and were eligible for inclusion in this systematic review. Only one was considered deficient.

## Discussion

This review’s objective was to identify the effects of amygdala neuromodulation on disruptive behavior in patients with intermittent explosive disorder refractory to conventional treatment. All of the studies involved ablation except Sturm et al.^22^ The behavior of three-fourths of the patients in these studies improved,^4^,^5^,^8^,^12^-^22^ although it should be noted that some studies reported no behavior changes^4^,^12^-^19^ or worsened behavior after surgery.^12^,^14^,^17^ More studies were from the United States than any other country, which may be related to its advanced neuropharmacological research facilities, significant funding for neuroscientific research, and clinical infrastructure that supports innovative neurosurgical interventions.

The study of aggressive behavior in response to certain stressful stimuli dates back to the end of the 19th century with experimentation on canines, which, after their temporal lobe was resected, became more docile and calmer in a process called “domestication.”^20^ In 1939, another study confirmed that temporal lobectomy with amygdalotomy in monkeys and cats produced the same effect.^23^ These findings, along with 1937 observations by Papez^24^ about the importance of the temporal lobe’s connection to the limbic system and its mediation of some emotions, motivated Narabayashi et al.^4^ to introduce ablative amygdalotomy as a treatment for severe and refractory aggressive behavior in humans.

More recently, an optogenetics study by Fritz et al.^6^ reported that the posterior ventral segment of the medial part of the amygdala is essential in intermittent explosive disorder. These authors also found this region has a close relationship with the ventromedial prefrontal cortex and the anterior cingulate cortex, which could explain the appearance of aggressive behavior in individuals with reduced connectivity between these structures.^6^

The studies in this review that obtained positive effects had a long follow-up period, which indicates that good results are long-term and are maintained in most patients.^4^,^5^,^8^,^12^-^22^ However, most of these studies were conducted 50-60 years ago. Despite these promising findings for individuals with a severely disabling pathology that is demanding for caregivers, neuropharmacological advances over the last 3 decades and the stigmatization of ablative psychosurgery have led to a significant decrease in amygdalotomies worldwide.^21^

In their original case series, Narabayashi et al.^4^ examined 60 patients with severe and refractory aggression who underwent amygdalotomy with stereotaxy, finding marked reduction in symptoms and high adaptation to the social environment in 85% of patients. However, only 40 patients were followed up long-term,^5^ of whom 67% continued to show significant symptomatic improvement approximately 3 years after the intervention. They observed that younger patients (between 5 and 13 years of age) presented better results than older ones. The same findings were observed for patients with comorbid epilepsy.

In a case series of 100 patients, Balasubramaniam & Ramamurthi^13^ found that post-epileptic patients had better outcomes than their counterparts. This stands out, since both Papez^24^ and Kluver & Bucy^23^ reported a relationship between certain forms of epileptic activity and emotional disorders, suggesting connections with seizure control and aggressiveness.

Approximately 30% of the patients experienced seizures, which were classified as grand mal, petit mal, psychomotor episodes, or temporal lobe episodes. Of these, approximately 40% resolved or significantly improved in frequency and severity, both clinically and in electroencephalogram results.^4^,^5^,^8^,^12^-^22^ However, studies with more evidence are needed to support this approach.

Some of the authors highlighted the effects of symptomatic improvement on the patient’s social and work environment, suggesting that stereotaxic ablative amygdalotomy could increase helpfulness at home and at work.^5^,^12^,^13^,^15^

Intermittent explosive disorders include aggressiveness, self-mutilation, hyperkinesia, pyromania, hostility, hypersexuality, and risk behaviors.^4^,^5^,^8^,^12^-^22^ In some studies, the type of aggressiveness pattern is discriminated, showing superior improvement in certain aspects. Something similar occurred in relation to intellectual disability, since some studies reported worse results in cases of comorbid severe intellectual disability.^5^,^12^,^15^

Some of the cited studies created their own scales, using cutoff points based on triggers of different intensities of disruptive behavior.^4^,^5^,^12^,^13^,^15^-^18^,^22^ However, few applied standardized scales currently approved by international regulations, such as the Modified Overt Aggression Scale, the Cattell 16PF Questionnaire, or the Hargreaves Nursing Rating Scale.^14^,^20^

No major differences were found between bilateral and unilateral stereotactic amygdalotomy.^4^,^5^,^8^,^12^-^22^ Ablation was unilateral in < 10% of the patients and was promoted in cases with comorbid infantile hemiplegia syndrome and focal seizures.^2^ Regarding the latter, laterality is defined according to seizure semiology or electroencephalographic or stereoelectroencephalographic findings. However, the evidence is insufficient to recommend unilateral or bilateral treatment in this context.^5^,^12^,^18^ Moreover, there was no homogeneity in the coordinates used to define the therapeutic target, varying between different nuclei and anatomical references, as well as between lesion induction methods, e.g., radiofrequency, diathermy, cryoablation, or wax or oil injection.^4^,^8^,^12^,^13^,^15^,^16^,^18^,^20^-^22^

Most studies (60%) reported no complications, although some reported unusual transient or permanent hemiparesis, hypersexuality, or language and memory changes, as well as subtle deficits in face recognition and de novo epilepsy.^4^,^14^-^18^

Finally, although stereotactic amygdalotomy generally had a positive effect on patient behavior, it cannot be stated that it is recommended for all patients with intermittent explosive disorder, since updated studies and more powerful epidemiological evidence are lacking. The last published cohort study was by Garcia-Muñoz et al.^25^ in 2019, but it was not included in our review because it had two neuromodulation targets, the amygdala and the posteromedial hypothalamus. That study included a group of 12 patients who underwent unilateral ablative therapy in both targets, finding a favorable response for refractory aggressiveness over 36 months of follow-up.^25^ Similarly, other systematic reviews have addressed this same issue, such as Gouevia et al.,^1^ in 2019, which included 27 articles on amygdalotomy as a treatment for aggressiveness. More than two-thirds of the those articles were not considered for the present review because they had at least one other simultaneous target or involved additional surgeries, which do not allow for a homogeneous evaluation of the results (e.g., frontal lobotomy, leucotomy, subcaudate tractotomy, cingulectomy, thalamotomy, fornicotomy, hippocampotomy, or anterior capsulotomy).^1^ Our study is the first systematic review to accurately and objectively document the effects of amygdalotomy on aggressiveness, as well as its positive effects on seizure control.

The posteromedial hypothalamus is currently the most studied and widely used target for treating refractory aggression, largely due to the contributions of Sano et al.^26^ and Messina et al.^27^ This target has demonstrated favorable outcomes not only for ablation as well as DBS. These positive results are evident in various case series, including one by Escobar-Vidarte et al.^28^ in our local context in 2021.

However, the good response achieved by targeting the amygdala for ablative or neurostimulation therapy for severe and refractory aggression, in addition to the positive effects on comorbid refractory epilepsy,^29^ makes this a promising field for future research. It will be necessary to determine whether this technique, which includes the possibility of calibration and reversibility, provides safe and potentially adjustable results, as were reported in perhaps the only published study to date on DBS of the amygdala for aggression management. In 2012, Sturm et al.^22^ reported on the case of a patient with autism spectrum disorder and refractory aggression who presented clinical improvement in both pathologies after DBS of the basolateral nucleus of the amygdala.^24^ Encouraging results were also found decades ago in rodent studies.^30^

Concomitant advances in connectomics, which identifies anatomical and functional neural networks in various brain circuits, should not be overlooked. Yan et al.^31^ used tractography and functional magnetic resonance imaging to perform a connectomic analysis of eight patients who underwent DBS electrode implantation in the nucleus accumbens, globus pallidus internus, anterior limb of the internal capsule, posterior hypothalamus, ventral capsule/ventral striatum, and basolateral amygdala. This analysis revealed a common network among these targets, which is implicated in the initiation, maintenance, and suppression of aggression. This study showed that provocative sensory, visual, and auditory stimuli cause the amygdala to initiate and specifically modulate aggressive behavior. Extensive connections to the hypothalamus and insular cortex activate the hypothalamic-pituitary axis and autonomic centers, preparing the body for an aggressive response. Additionally, reinforcement-based decision-making processes in the nucleus accumbens and insular cortex, along with the ventromedial, orbitofrontal, and cingulate cortices, are responsible for modulating and/or suppressing aggressive behavior, as observed in patients with self-aggressive behaviors in autism spectrum disorder.^22^ This analysis suggests that DBS may have a neuromodulatory effect on this network.^31^

In conclusion, although ablation or DBS of the amygdala has a positive effect on patient behavior and seizure control, these techniques cannot, as of yet, be recommended for cases of extreme aggressive behavior because most of the included studies were published more than 30 years ago, with only one included case series having been published recently. Thus, further validation of these results is necessary.

## Disclosure

The authors report no conflicts of interest.

## Figures and Tables

**Table 1:** General characteristics of the included studies

Author (country)	Patient type/comorbidities	Age(years)	Sample size n (% male)	Laterality/surgical target	Surgical technique	Adverse effects
Narabayashi et al.^4^ (Japan)	Behavioral disorders in patients with a history of epilepsy (39), post-encephalitis (6), mental retardation (11), posttraumatic (1), psychopathic disease (1), tuberous sclerosis (2).	5-35	60 (63.3)	Uni- (39) or bilateral (21) stereotaxic amygdalotomy: amygdaloid nucleus to the anterosuperior border of the temporal horn and the lower margin of the nucleus, located a few mm above the tip of the temporal horn.	A 0.6-0.8 mL injection of oil and wax + lipiodol produced a lesion 8 to 10 mm in diameter.	Slight hypersexuality lasting for only a few days after operation (1).
Heimburger et al.^12^ (United States)	Behavioral disorders (hyperactivity, hostility, aggressiveness, destructiveness, and hypersexuality) and a history of epilepsy (16), and mental retardation (17).	7-61	26 (57.7)	Unilateral (16/24) or bilateral (8/24). Stereotactic amygdalotomy (target point: 2.0 cm lateral to the midline, 1.0 cm below the intercommissural line).	Cryoprobe used for 5 min at -120 °C; for the second lesion, the probe was withdrawn 5 mm and the area was cooled again to -120 °C for 3 min. Lesion size estimate: 8 to 10 mm in diameter.	N/R
Balasubramaniam & Ramamurthi^13^ (India)	Mental and behavioral disorders (assaultive, self-destructive, destructive, hyperkinetic, pyromania, hyperoral tendency, wandering tendency)/schizophrenia (3), epilepsy (43), encephalitis (37), perinatal damage (3), and cerebral agenesis (14).	0-50	100 (82.0)	Bilateral (87) or unilateral (13). Stereotactic amygdalotomy (4 mm in front of temporal horn in lateral view and 22 mm from the midline in anteroposterior view).	Diathermy coagulation (48), mechanical destruction with loop (29), and injection of wax or oil (31). Lesion size: 200-1,800 mm^3^.	N/R
Narabayashi & Mizutani^5^ (Japan)	Behavioral disorders (hyperactive, destructive) in patients with a history of epilepsy (25)	N/R	25 (N/R)	Unilateral (8/12) or bilateral (4/12) stereotaxic amygdalotomy. Lesion target not reported.	N/R	N/R
Hitchcock & Cairns^14^ (Scotland)	Behavioral disorders (rebellion, hyperactive, destructive) and a history of epilepsy (16), and mental retardation (5).	8-46	18 (72.2)	Bilateral radiofrequency amygdalotomy (18). Lesion target not reported.	N/R	Hemiparesis (1), face recognition (2).
Kiloh et al.^15^ (Australia)	Disabling aggressive behavior (assaultive, self-destructive, destructive, hyperkinetic, pyromania, hyperoral tendency, wandering tendency)/epilepsy (5), mental retardation (13), schizophrenia (4), and personality disorder (6).	13-37	18 (77.8)	Unilateral (3) or bilateral (15) radiofrequency amygdalotomy (5 mm medial to the medial border of the lateral cleft).	12 bipolar patients received radiofrequency at 60-65 °C for 30 s, three patients received Cooper cryoprobe at -700 °C for 3 min, and three patients received Cooper cryoprobe at -1,200 °C for 3 min. Lesion diameter not reported.	Permanent hemiparesis (1), epilepsy in non-epileptic patients (4).
Balasubramaniam & Kanaka^16^ (India)	Behavioral disorders/ schizophrenia (3), epilepsy (80), encephalitis (81), perinatal damage (11), cerebral agenesis (35), and schizophrenia (12).	N/R	235 (N/R)	Unilateral (28) or bilateral (207) stereotaxic amygdalotomy. Lesion target not reported.	Lesions produced through wax injection or diathermy coagulation. Lesion diameter not reported.	Transient (6) or permanent (2) hemiplegia.
Heimburger et al.^17^ (United States)	Behavioral disorders (aggressiveness, rage attack, hyperactivity, destructive) and a history of epilepsy and depression/epilepsy (49), head injury (22), and febrile illness (14).	8-61	58 (N/R)	Unilateral (45) or bilateral (13) stereotactic amygdalotomy: 5 mm superior and 5 mm anterior to the tip of the temporal horn.	N/R	Temporary memory loss (7), decrease of peripheral vision (5), increased sexual desire (3), hemiparesis (1), and language impairment (1).
Ramamurthi^18^ (India)	Behavioral disorders: physical aggression, hyperkinesis, pyromania, destructive, and self-destructive tendencies/N/R.	N/R	481 (N/R)	Bilateral stereotactic amygdalotomy: the coordinates were calculated after demonstrating the tip of the temporal horn by ventriculography.	The lesions, produced through a combination of diathermy and myodil wax, were approximately 600-900 mm^3^ in size.	In 28 patients (6%), there was a slightly delayed hemiplegia, which recovered over a few weeks.
Hood et al.^19^ (Switzerland)	Patients with complex partial seizures accompanied by aggressive outbursts/epilepsy (4), post-encephalitis (2), posttraumatic stress (2), and febrile illness (1).	17-57	4 (100.0)	Unilateral stereotactic amygdalotomy (4): lesion target not reported.	N/R	N/R
Gouveia et al.^20^ (Brazil)	Patients with intractable aggressive behavior that put their lives at risk and interfered with their social interaction, autism spectrum cognitive disability/autism (3), and mental retardation (4).	19-32	4 (75.0)	Bilateral stereotactic amygdalotomy (4): lesion target not reported.	Radiofrequency was applied at 80 °C for 90 s.	N/R
Lee et al.^8^ (United States)	Case 1: patient with partial complex seizures (controlled with medication) and severe behavioral changes (assaultive and self-destructive) after viral encephalitis. Case 2: patient with partial complex seizures controlled with medications and uncontrollable aggressiveness (assaultive and self-destructive) after chickenpox encephalitis/epilepsy (2) and viral encephalitis (2).	19 and 21	2 (100.0)	Bilateral stereotactic amygdalotomy (2) (20-25 mm right, 18-23 mm left of middle line).	Radiofrequency at 80 °C for 90 s. Lesion size was 15 mm in diameter in both patients.	N/R
Fountas et al.^21^ (United States)	Patient with a chronic history of psychiatric disorder (self-mutilation) and resistance to medical and behavioral treatment.	18	1 (0.0)	Bilateral stereotactic amygdalotomy: the plane coordinates for the target points on the right side were x = 83.0, y = 98.4, z = 123.4; target points on the left side were x = 124.4, y = 98.9, z = 121.8.	Three radiofrequency lesions, applied at 90 °C for 90 s.	N/R
Sturm et al.^22^ (Germany)	Patient with early-childhood autism, mental retardation and SIB/autism.	14	1 (100.0)	Deep brain stimulation of amygdaloid nuclei and the supra-amygdaloid projection system: contact 0: border region between the ventral (paralaminar) and the BL nucleus. Contact 1: center of the BL nucleus. Contact 3: sublenticular territory - the striatopallidal amygdalofugal fibers, fibers related to the external capsule, and the uncinate fascicle, as well as the magnocellular cholinergic system of the forebrain and the extended amygdala.	Parameters: 120 μs, 130 Hz pulse-following frequency, and 2-6.5 volt amplitude. Voltage was slowly increased over the course of 12 months.	N/R

N/R = not reported; SIB = self-injurious behavior.

**Table 2:** Outcome variables obtained from the included studies

Author	Aggression assessment scale	Aggressiveness results	Epilepsy results	Quality of life	Follow-up time
Case series					
Narabayashi et al.^4^	Authors’ own scale: A) Great improvement; B) Moderate improvement; C) Mild improvement; D) No change.	A) 29 (48%); B) 22 (37%); C) 7 (12%); D) 2 (3%). 68 (97%) patients showed improvement.	Seizures improved in 19/46 patients. EEG changes: 5 spikes abolished, 10 spikes reduced, and 4 spikes decreased.	Patients became more obedient, calm, and even educable. They could adapt to the demands of daily life. In some cases, the improvement in attitude and cooperation was such that the patient could enjoy school life as a result of the surgery. One of the patients also improved to the point of being able to care for his younger siblings.	6-48 months
Heimburger et al.^12^	Authors’ own scale: +++ = severe behavioral anomaly; ++ = moderate; + = mild; 0 = none	Behavioral abnormalities were eliminated in 7 (35%) patients and markedly improved in 9 (45%), i.e. 80% overall improvement. Aggression worsened in 1 (5%) patient and no change occurred in and 3 (15%).	Seizures eliminated in 4 (19%) patients and appreciably improved in 12 (57%) patients. Seizures improved in 16 (76%) patients.	The changes led to greater medication control and decreased institutionalization.	> 2 years
Balasubramaniam & V Ramamurthi^13^	Authors’ own scale: a) Patient does not need further medication. The patient mixes easily with others; b) Very docile with only occasional episodes of aggression; c) Manageable through medication, but this does not lead to a useful life; d) Temporary improvement followed by relapse; e) No change; f) Died.	a) 6 (6%);b) 33 (33%); c) 36 (36%); d)12 (12%); e) 4 (4%); f) 9 (9%).	Post-epileptic symptoms occurred in 43 (43%) patients, of whom 32 (75%) are adequately controlled. No changes reported in seizure pattern, frequency, or intensity. No EEG changes.	Two patients committed to mental institutions improved so much that they could be released. Patients became more obedient and more interested in their surroundings. One patient began to learn to read and developed an interest in music.	1-4 years
Narabayashi & Mizutani^5^	Authors’ own scale: A) Marked behavioral and emotional improvement in the patient, who can adapt to the social environment according to his/her intellectual level; B) Adaptation to the family environment only; C) Slight symptom improvement.	A) 48% (12); B) 40% (10); C) 12%(3); D) 0%.	Seizures were eliminated in 9 (36%) patients, reduced in 8 (32%) patients, and no change occurred in 7 (28%) patients. Seizures improved in 17 (68%) patients.	Five cases with category A results showed extreme improvement and were considered to have normal behavior and satisfactory social adaptation (e.g., entering school and/or getting a job).	1-6 years
Hitchcock & Cairns^14^	Cattell’s 16PF questionnaire, the Hargreaves Nursing Rating Scale, and adaptive behavior scales.	Postoperative improvement in 6 (33%) patients, 4 of whom showing high overall improvement; no real change in the other 11 (60%) patients; symptoms worsened in only 1 (5%) patient.	Seizures occurred in 16 patients. The frequency and severity of fits were notably reduced in 3 patients.	Of patients previously incapable of any form of occupational activity, 4 (of 9) are now either employed or attending occupational therapy units.	5 years
Kiloh et al.^15^	Authors’ own scale: Marked improvement; Slight improvement; No change.	Marked improvement in 4 (22%) patients; slight improvement in 5 (27%) patients; no change in 9 (50%) patients; 2 patients had slight improvement and returned to baseline state.	Seizures occurred in 5 (27%) patients: 4 with generalized tonic-clonic seizures, and 1 with psychomotor attacks. No changes reported in seizure pattern, frequency, or intensity. No EEG changes.	Marked improvement occurred in some cases: they exhibited less frequent and/or less intense aggression or self-mutilation to such an extent that their lives changed drastically and the attitudes of their immediate associates changed toward them.	2-5 years
Balasubramaniam & Kanaka^16^	Authors’ own scale: A) No medication needed - the patient can easily mix with others; B) The patient is mainly docile and only presents occasional outbreaks; C) Patient is manageable when medicated even though he/ she does not have a useful life; D) Transient improvement but relapse; E) No change; F) Died.	A) 14 (5.9%); B) 74 (31%); C) 90 (38%); D) 43 (18%); E) 5 (2%); F) 9 (3.8%).	Post-epileptic symptoms occurred in 80 (34%) patients. No changes reported in seizure pattern, frequency, or intensity. No EEG changes.	N/R	N/R
Heimburger et al.^17^	Authors’ own scale: Improvement; No change; Worsening.	Improvement occurred in 17 (38%) patients, no change in 25 (56%) patients, and worsening in 2 (4%) patients.	Improvement occurred in 21 (43%) patients; no change occurred in 27 (55%) patients, and worsening occurred in 1 (2%) patient.	N/R	1-11 years
Ramamurthi^18^	Authors’ own scale: Excellent; Good; Moderate; No improvement.	Good improvement maintained in 264 (55%) patients; moderate improvement maintained in 72 (15%) patients, and 144 (30%) patients did not respond to surgery.	N/R	N/R	> 3 years
Hood et al.^19^	N/R	Significant improvement in 3 (75%) patients (one died in a car accident 6 months after surgery); no improvement in 1 (25%) patient.	No seizures in 2 patients, marked improvement in 1 patient, and 1 patient remained unchanged.	N/R	6-45 months
Gouveia et al.^20^	OAS	Aggressive behavior reduction: Case 1) 84.6%; Case 2) 80%; Case 3) 88.9%; Case 4) 56.2%.	N/R	All changes resulted in notable behavioral alterations that alleviated suffering and led to improved quality of life.	3 years
Case reports					
Lee et al.^8^	Psychophysiological evaluations	The number of aggressive events decreased in both patients, but both continued to have difficulty controlling them. The results were considered in unsuccessful in 1 patient (50%).	Seizures were controlled before the surgery. No changes in seizure pattern, frequency, or intensity. No EEG changes.	No significant quality of life changes occurred. One patient was discharged from a state hospital to a personal care home. He has lived there without an assaultive outburst for 7 months. The other patient assaulted the supervisor of his personal care home and was imprisoned until the following summer.	8 years
Fountas et al.^21^	N/R	The patient was calm and sociable. No episodes of self-mutilation or other aggressive behavior was reported for 18 months. At that time, he experienced an acute episode of violent blows to the head.	N/R	N/R	1.5 years
Sturm et al.^22^	The father developed an empirical score: 1) No restraint necessary; 2) Restraint of the wrists suffices and is well tolerated; 3) Restraint of the wrists prevents self harm in an almost adequate manner; 4) Restraint of the wrists fails to completely control SIB; 5) Restraint cannot control SIB but skin lesions do not occur; 6) Restraint does not prevent skin lesions or life threatening self-injury.	Before battery depletion, the father rated his son’s behavior with scores of 2-3, but these scores continuously rose to 6 in the period without stimulation. One and 6 weeks after re-starting DBS, the scores dropped to 2 and 1-2 (no restraint required), respectively.	N/R	Core autistic spectrum symptoms (fear and anxiety, emotional tensions, sleep disorder, impaired self-regulatory processes in response to auditory and visual stimuli, impaired affect modulation, and impaired communicative eye-contact) improved gradually and have now reached their lowest levels.	26 months

DBS = deep brain stimulation; EEG = electroencephalography; N/R = not reported; OAS = Overt Aggression Scale; SIB = self-injurious behavior.

**Table 3:** Results of the study quality assessment using Murad et al.’s instrument

Author	1	2	3	4	5	6	Total	Quality
Case series								
Narabayashi et al.^4^	1	1	1	0	1	1	5	Good
Heimburger et al.^12^	0	1	0	1	1	1	4	Acceptable
Balasubramaniam & Ramamurthi^13^	0	1	1	1	1	1	5	Good
Narabayashi & Mizutani^5^	0	0	1	1	1	0	3	Acceptable
Hitchcock & Cairns^14^	1	1	1	1	1	1	6	Good
Kiloh et al.^15^	0	1	1	1	1	1	5	Good
Balasubramaniam & Kanaka^16^	0	1	1	1	1	0	4	Acceptable
Heimburger et al.^17^	0	1	1	1	1	1	5	Good
Ramamurthi^18^	0	1	1	1	1	1	5	Good
Hood et al.^19^	0	1	0	1	0	0	2	Poor
Gouveia et al.^20^	1	1	1	1	1	1	6	Good
Case reports								
Lee et al.^8^	0	1	0	1	1	0	3	Acceptable
Fountas et al.^21^	0	1	0	1	1	1	4	Acceptable
Sturm et al.^22^	0	1	1	1	1	1	5	Good

## Data Availability

The data that support the findings of this study are available from the corresponding author upon reasonable request.
