# Supplementary material for: The amygdala as a therapeutic target for aggressive and disruptive behaviors: a systematic review
Source: Braz J Psychiatry. 2024 Nov 25;46:e20243582. doi: 10.47626/1516-4446-2024-3582 (PMC11773330; doi:10.47626/1516-4446-2024-3582)
Supplement: Supplementary file 1 [file bjp-46-e20243582-suppl1.pdf]

http://doi.org/10.47626/1516-4446-2024-3582
The amygdala as a therapeutic target for aggressive and disruptive behaviors: a systematic review – Salcedo-Moreno et al.

Supplementary Box S1 Search equation used in each database

**Database**	**Search term**
SCOPUS	((TITLE-ABS-KEY (aggressivity) OR TITLE-ABS-KEY (aggression) OR TITLE-ABS-KEY ("aggressive behavior") OR TITLE-ABS-KEY ("aggressive behaviour") OR TITLE-ABS-KEY ("aggressive behaviors") OR TITLE-ABS-KEY ("behavioral disorders") OR TITLE-ABS-KEY ("behavior disorders") OR TITLE-ABS-KEY ("disruptive behavior") OR TITLE-ABS-KEY ("violent behavior") OR TITLE-ABS-KEY ("violent behaviour") OR TITLE-ABS-KEY ("explosive disorder") OR TITLE-ABS-KEY ("aggressiveness"))) AND ((TITLE-ABS-KEY ("psychosurgery") OR TITLE-ABS-KEY ("neurosurgical treatment") OR TITLE-ABS-KEY ("stereotactic") OR TITLE-ABS-KEY ("stereotaxic") OR TITLE-ABS-KEY ("amygdalotomy") OR TITLE-ABS-KEY ("neuronavigation") OR TITLE-ABS-KEY ("radiosurgery") OR TITLE-ABS-KEY ("amygdalectomy") OR TITLE-ABS-KEY ("psycho surgery") OR TITLE-ABS-KEY (dbs) OR TITLE-ABS-KEY (" deep brain stimulation")))
MEDLINE	((((((((((("aggressivity"[Title/Abstract]) OR ("aggression"[Title/Abstract])) OR ("aggressive behavior"[Title/Abstract])) OR ("aggressive behaviour"[Title/Abstract])) OR ("aggressive behaviors"[Title/Abstract])) OR ("behavioral disorders"[Title/Abstract])) OR ("behavior disorders"[Title/Abstract])) OR ("disruptive behavior"[Title/Abstract])) OR ("violent behavior"[Title/Abstract])) OR ("violent behaviour"[Title/Abstract])) OR ("explosive disorder"[Title/Abstract])) OR ("aggressiveness"[Title/Abstract])) AND ((((((((((("psychosurgery"[Title/Abstract]) OR ("neurosurgical treatment"[Title/Abstract])) OR ("stereotactic"[Title/Abstract])) OR ("stereotaxic"[Title/Abstract])) OR ("amygdalotomy"[Title/Abstract])) OR ("deep brain stimulation"[Title/Abstract])) OR ("neuronavigation"[Title/Abstract])) OR ("radiosurgery"[Title/Abstract])) OR ("amygdalectomy"[Title/Abstract])) OR ("psycho surgery"[Title/Abstract])) OR ("dbs"[Title/Abstract])) OR ("deep brain stimulation"[Title/Abstract]))
WEB OF SCIENCE	1. TEMA: (Aggressivity) OR TEMA: (Aggression) OR TEMA: ("Aggressive behavior") OR TEMA: ("Aggressive behaviour") OR TEMA: ("Aggressive behaviors") OR TEMA: ("Behavioral disorders") OR TEMA: ("Behavior disorders") OR TEMA: ("Disruptive behavior") OR TEMA: ("Violent behavior") OR TEMA: ("Violent behavior") OR TEMA: ("Explosive disorder") OR TEMA: (Aggressiveness)<br>2. TEMA: (Psychosurgery) OR TEMA: ("Neurosurgical treatment") OR TEMA: (Stereotactic) OR TEMA: (Stereotaxic) OR TEMA: (Amygdalotomy) OR TEMA: (Amygdalectomy) OR TEMA: ("Deep brain stimulation") OR TEMA: (Neuronavigation) OR TEMA: (Radiosurgery) OR TEMA: ("dbs")<br>3. #2 AND #1
OVID EBMR COCHRANE	EBM Reviews - Cochrane Database of Systematic Reviews <2005 to May 15, 2024><br>EBM Reviews - ACP Journal Club <1991 to April 2024><br>EBM Reviews - Database of Abstracts of Reviews of Effects <1st Quarter 2016><br>EBM Reviews - Cochrane Clinical Answers <April 2024><br>EBM Reviews - Cochrane Central Register of Controlled Trials <April 2024><br>EBM Reviews - Cochrane Methodology Register <3rd Quarter 2012><br>EBM Reviews - Health Technology Assessment <4th Quarter 2016><br>EBM Reviews - NHS Economic Evaluation Database <1st Quarter 2016><br><br>1 Aggressivity.mp. [mp=ti, ot, ab, tx, kw, ct, sh, fx, hw] 43<br>2 Aggression.mp. [mp=ti, ot, ab, tx, kw, ct, sh, fx, hw]4631<br>3 "Aggressive behavior".mp. [mp=ti, ot, ab, tx, kw, ct, sh, fx, hw] 772<br>4 "Aggressive behaviour".mp. [mp=ti, ot, ab, tx, kw, ct, sh, fx, hw] 327<br>5 "Aggressive behaviors".mp. [mp=ti, ot, ab, tx, kw, ct, sh, fx, hw] 197<br>6 "Behavioral disorders".mp. [mp=ti, ot, ab, tx, kw, ct, sh, fx, hw] 525<br>7 "Behavior disorders".mp. [mp=ti, ot, ab, tx, kw, ct, sh, fx, hw] 1993<br>8 "Disruptive behavior".mp. [mp=ti, ot, ab, tx, kw, ct, sh, fx, hw] 981<br>9 "Violent behavior".mp. [mp=ti, ot, ab, tx, kw, ct, sh, fx, hw] 157<br>10 "Violent behavior".mp. [mp=ti, ot, ab, tx, kw, ct, sh, fx, hw] 157<br>11 "Explosive disorder".mp. [mp=ti, ot, ab, tx, kw, ct, sh, fx, hw] 43<br>12 Aggressiveness.mp. [mp=ti, ot, ab, tx, kw, ct, sh, fx, hw] 668<br>13 1 or 2 or 3 or 4 or 5 or 6 or 7 or 8 or 9 or 10 or 11 or 12 8073<br>14 Psychosurgery.mp. [mp=ti, ot, ab, tx, kw, ct, sh, fx, hw] 37<br>15 "Neurosurgical treatment".mp. [mp=ti, ot, ab, tx, kw, ct, sh, fx, hw] 69<br>16 Stereotactic.mp. [mp=ti, ot, ab, tx, kw, ct, sh, fx, hw] 2887<br>17 Stereotaxic.mp. [mp=ti, ot, ab, tx, kw, ct, sh, fx, hw]313<br>18 Amygdalotomy.mp. [mp=ti, ot, ab, tx, kw, ct, sh, fx, hw] 1<br>19 "Deep brain stimulation".mp. [mp=ti, ot, ab, tx, kw, ct, sh, fx, hw] 1755<br>20 Neuronavigation.mp. [mp=ti, ot, ab, tx, kw, ct, sh, fx, hw] 397<br>21 Radiosurgery.mp. [mp=ti, ot, ab, tx, kw, ct, sh, fx, hw] 1449<br>22 dbs.mp. [mp=ti, ot, ab, tx, kw, ct, sh, fx, hw] 1700<br>23 Amygdalectomy.mp. [mp=ti, ot, ab, tx, kw, ct, sh, fx, hw] 1<br>24 14 or 15 or 16 or 17 or 18 or 19 or 20 or 21 or 22 or 23 5943<br>25 13 and 2425
